# Protocol for measuring the Young’s modulus of organoids using atomic force microscopy

**DOI:** 10.1016/j.xpro.2025.103825

**Published:** 2025-05-20

**Authors:** Tianzhen Zhang, Lina Ma, Shen Ling, Yupeng Chen, Zhongtao Zhang, Dan Tian, Yingchi Yang

**Affiliations:** 1 Department of General Surgery, Beijing Friendship Hospital, Capital Medical University, Beijing 100050, China; 2 State Key Lab of Digestive Health, Beijing Friendship Hospital, Capital Medical University, Beijing 100050, China; 3 National Clinical Research Center for Digestive Diseases, Beijing 100050, China; 4 Immunology Research Center for Oral and Systemic Health, Beijing Friendship Hospital, Capital Medical University, Beijing 100050, China

**Keywords:** Biophysics, Atomic force microscopy, AFM, Cancer, Organoids

## Abstract

Atomic force microscopy (AFM) is extensively applied to measure cell and tissue mechanics but lacks a standardized organoid stiffness assessment. Here, we present a protocol for quantifying the Young’s modulus of organoids via AFM, combining force-curve analysis with an optimized probe. We describe steps for preparing organoids, OCT embedding, slicing, AFM detection, and force-curve analysis. By mechanically addressing gaps in organoids, this protocol improves reproducibility and expands the capabilities of biomechanical research.

## Highlights

• Steps to perform atomic force microscopy on organoids
• Instructions for embedding organoids with OCT gel
• Guidance on analyzing AFM-measured organoid curves using Hertz contact models

## Abstract (editor highlights)

Publisher’s note: Undertaking any experimental protocol requires adherence to local institutional guidelines for laboratory safety and ethics.

## Graphical abstract

**Figure:**
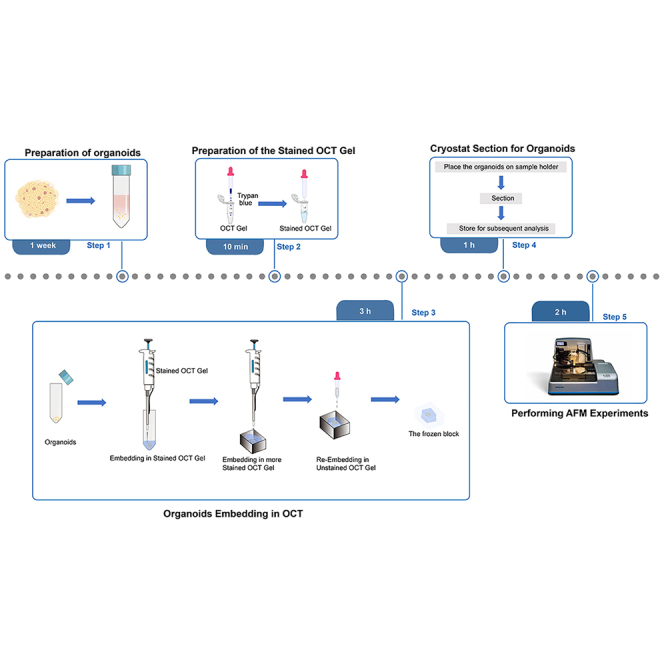

## Before you begin

Select patients diagnosed with colorectal cancer for this study, ensuring that informed consent is obtained from all participants before their involvement.

### Institutional permissions

The organoid cultures derived from the tumor tissues of participating patients, along with the AFM experiments, are approved by the hospital’s ethics committee. For the culture of other cell types, such as tumor cells from various cancer types or cells isolated from mice or other vertebrates, appropriate ethical or animal welfare approvals must be obtained before commencing experimentation.

**Figure 1:**
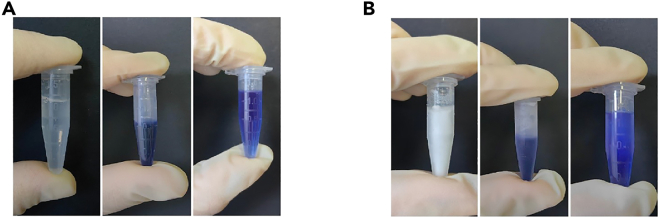
The state of Trypan Blue and OCT at varying temperatures (A) From left to right are the unstained OCT, trypan blue, and trypan blue-stained OCT at room temperature. (B) From left to right are the unstained OCT, trypan blue, and trypan blue-stained OCT at −80°C.

**Figure 2:**
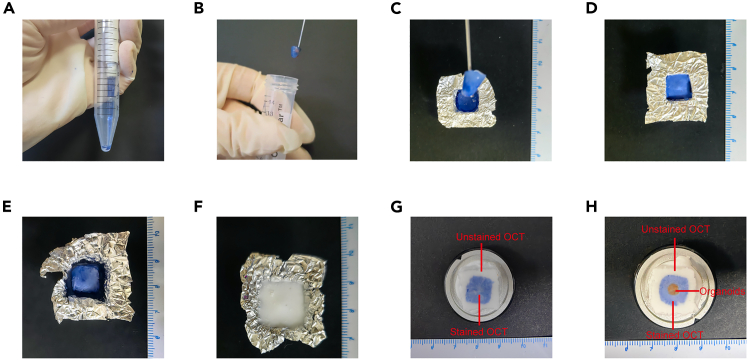
Steps for Embedding Organoids in OCT (A) Embedding in stained OCT gel. (B) Transfer the frozen OCT block. (C) Embedding in stained OCT gel in a small tin box (0.8 cm^3^). (D) Frozen block image after initial embedding. (E) Re-Embedding in unstained OCT gel in a big tin box (1.2 cm^3^). (F) Frozen block image after re-embedding. (G) Image cut Not reaching the organoids level to the organoids level. (H) Image cut reaching the organoids level to the organoids level.

**Figure 3:**
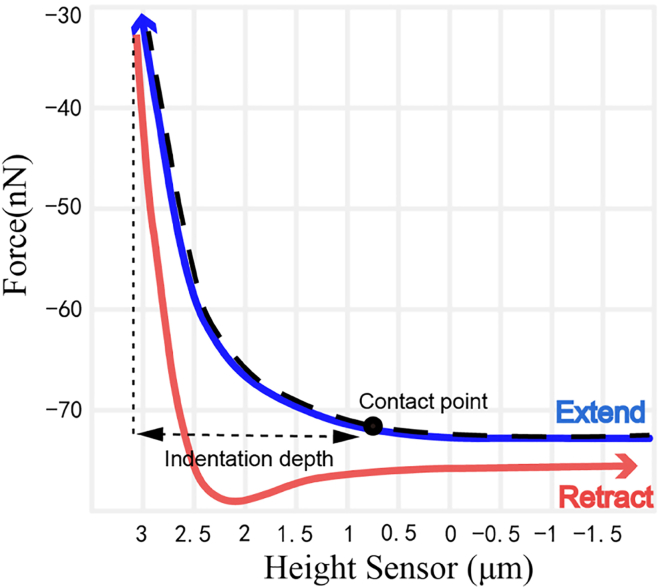
High-Quality Force Curve

### [Preparation 1] Preparation of organoids

**Timing: 1 week**

1. Prepare organoids:
  a. Excise a 1 cm^3^ tumor tissue sample surgically.
  b. Promptly immerse in a tissue preservation solution.
  c. Store the sample at 4°C for transportation to the laboratory.
  d. Upon arrival, process the tissue for organoid culture.

***Note:*** All organoids are obtained via a standardized culture protocol provided by the organoid company, and successful acquisition of organoids is achieved within 1 week.

### [Preparation 2] Preparation of stained OCT gel

**Timing: 10 min**

2. Add 100 μL of trypan blue to 300 μL of OCT compound, and mix thoroughly through repeated pipetting. The resulting mixture serves as a labeling agent for organoids (Figure 1).

## Key resources table

**Table:** 

REAGENT or RESOURCE	SOURCE	IDENTIFIER
**Chemicals, peptides, and recombinant proteins**		

OCT compound	Sakura	Cat# 4583
Phosphate-buffered saline	Solarbio	Cat# P1020
Trypan blue solution	Sigma	Cat# T8154

**Software and algorithms**		

Nanoscope instrument software	Nanoscope Instruments	Nanoscope AFM software

**Other**		

Glass coverslip	Citotest	Cat# 80312-8181
AFM	Bruker	DIMENSION icon with Scan Asyst
AFM setup	Research NanoScope	Research NanoScope 10.00
AFM cantilever	Bruker	SAA-SPH-10UM
Pipette	Thermo Scientific	Cat# 4641100N
Cryostat	Leica	Cat# CM1950

## Step-by-step method details

### Organoid embedding in OCT

**Timing: 3 h**

1. Sedimentation of organoids: Before embedding the organoids, allow them to settle at 4°C for 2 h, ensuring they concentrate at the bottom of the tube.
2. Extraction of sedimented organoids: Remove the preservation solution layer by layer, ensuring that the organoids remain at the bottom of the tube.
3. Embedding in stained OCT gel:
  a. Surround the organoids with the prepared stained OCT gel to ensure uniform coverage.
  b. Freeze the embedded organoids at −80°C for 5 min (Figure 2A).
4. Re-Embedding in OCT gel:
  a. Transfer the frozen OCT block (Figures 2B and 2C).
  b. Add unstained OCT gel for re-embedding (Figure 2D).
  c. Refreeze at −80°C for long-term storage.

***Note:*** The embedding process requires approximately 1 h.

### Cryostat section for organoids

**Timing: 1 h**

5. Apply a small amount of OCT compound to the sample holder, followed by positioning the embedded organoids (Figure 2E) onto it.
6. Freeze the sample holder at −20°C in cryostat for 3–5 min until the clear OCT gel sets (Figure 3F).
7. Start to sectioning:
  a. Set the cryostat thickness to 6 μm for initial sectioning.
  b. Identify organoids based on their distinct color patterns (blue OCT gel surrounded by white OCT gel) (Figure 2G).
  c. Once identified, adjust the thickness to 100 μm and prepare two continuous sections on separate slides^1^ (Figure 2H).

**CRITICAL:** 100 μm was determined to be the most suitable thickness for operational purposes and instrument detection. A thickness of 100 micrometers generally ensures the uniformity of mechanical properties in biological specimens, thereby minimizing significant local variations in mechanical characteristics that could potentially arise from excessively thin sections. This dimension aligns with the assumptions of the Hertzian Contact Model, which presumes the sample to be a homogeneous, isotropic semi-infinite body. The 100 μm thickness adequately satisfies this assumption, as the indentation depth of the probe is typically much smaller than the section thickness, thus ensuring the validity of the model.

8. Store the slides in a slide case for subsequent analysis.

**CRITICAL:** Transfer the frozen embedded organoids to the cryostat immediately after removing them from −80°C to minimize the risk of OCT gel melting at ambient temperature.

**CRITICAL:** Maintain consistency and precision in the cutting technique to preserve the quality of the profile.

### Performing AFM experiments on organoids in liquid environment

**Timing: 2 h**

9. Cantilever selection: Select the SAA-SPH-10 μm cantilever with a spring constant of approximately 0.1 N/m, suitable for measuring cellular stiffness ranging from hundreds of Pascals (Pa) to thousands of Pascals (kPa).

***Note:*** For this experiment, we utilized a cantilever from Bruker with a spring constant(K) of 0.158 N/m and a radius of curvature (ROC) of 10.25 μm.

10. Attach the probe to the probe holder and then mount the probe holder onto the scanner tube.
11. After seeding the organoids onto glass coverslips (as described in the previous section, “cryostat section for organoids”), carefully position the coverslip containing the organoids onto the AFM sample stage.
12. Immerse both the probe and organoids in a phosphate-buffered saline (PBS) solution.

***Note:*** Both cantilever calibration and the experiment are conducted within a PBS liquid environment.^2^^,^^3^

13. Align the laser beam with the cantilever so that the SUM value displayed on the photodetector reaches its maximum.
14. Adjust the VERT and HORIZ settings to bring both values close to zero:
  a. Calibrate the deflection sensitivity of the system using the thermal excitation method in the Nanoscope software. In our experiments, the deflection sensitivity is approximately 26–30 nm/V.
  b. Under atomic force microscopy, identify the precise position of the organoids and subsequently perform probe engage.
  c. Approach the surface of the organoids with the cantilever probe, applying a low-setpoint feedback force of 0.8 nN.
  d. Acquire the force curve to determine the optimal setpoint force value; the reasonable deformation range is 500–1000 nm.
    ***Note:*** This level of deformation ensures that the percentage strain in the organoids remains below 10%, thereby minimizing the effect of the bottom surface.^4^
  e. Conduct the experiments with a data acquisition rate of 1 Hz.
  f. Collect force curves using AFM, recording a total of 36 curves per sample.
    ***Note:*** Approximately 20 of these curves should be analyzable. Process and average these analyzable curves for further analysis.
    ***Note:*** Due to the consistent temperature and humidity maintained throughout the sample measurement process, the average value of Young's modulus remains unchanged even when the same sample is re-measured after an interval of 1 h. Consequently, the sequence of the detection procedures has no impact on the measurement outcomes.
    **CRITICAL:** The environmental control cabinet encapsulates or supports the AFM body, providing stable environmental conditions. It ensures the maintenance of constant temperature and humidity, thereby preventing thermal expansion or contraction of samples and AFM components due to environmental fluctuations. Measurements of the samples were performed in a liquid environment, thus eliminating the need to consider humidity effects, within a room-temperature liquid environment.
    **CRITICAL:** To verify the stability of organoids properties over this period, the initial set of experiments was repeated under similar conditions, and the results were analyzed comparatively. No significant changes in the average Young’s Modulus of the organoids were detected.
    **CRITICAL:** It is important to note that the optimal measurement duration may vary depending on the type of cells being studied. It is recommended to optimize the experimental timeline based on the specific characteristics of the cells under investigation.
    **CRITICAL:** The Hertzian Contact Model is only applicable to scenarios involving small deformations in elastic materials, such as biological tissues. This model assumes that the compression depth is substantially smaller than the sample thickness, generally less than 10% of the sample thickness. Excessive pressing depth may lead to plastic deformation or undermine the validity of the model.

## Expected outcomes

This protocol outlines a method for measuring the Young’s Modulus of organoids, including but not limited to organoids derived from colorectal cancer.

This protocol can also be applied to the mechanical property measurements of other cell types, such as organoids derived from other types of human tumors or non-tumor origins, as well as other non-tumor cells.

## Quantification and statistical analysis

### Measurement of Young’s modulus

1. Pre-processing the Raw Data.
  The raw force curves were processed using Nanoscope data analysis software for the purposes described below.
  a. Identify and select force curves that meet the criteria of the analysis (Figure 3).
  b. Baseline Calibration: Correction of slope artifacts in the force curve caused by drift during measurements.
  c. Data Export: Save the processed data in Excel file format for further analysis.
2. Calculating Young’s Modulus.

The formula for Young’s modulus is defined as follows:

(Equation 1) $$F_{ti_{P}}=\frac{4}{3}E^{*}\sqrt{Rd^{3}}+F_{a\mathrm{d}h}$$

Where: *F*_*tip*_:Total force applied by the tip on the sample. *E*^∗^: Effective Elastic Modulus. *R*: radius of curvature of the tip. *d*: Indentation Depth. *F*_adh_: Adhesion Force.

**CRITICAL:** a. Rationality of the Curve Shape Criteria: The force curve should exhibit a characteristic shape, including non-contact, contact, and adhesion zones. Significance: A characteristic force curve shape confirms that the interaction between the probe and the sample is normal and free from abnormal interference. b. Clarity of Contact Points Criteria: The contact point must be clearly defined, ensuring accurate determination of the moment when the probe makes contact with the sample. Significance: Clearly defined contact points serve as the basis for accurately calculating elastic modulus and deformation. c. Smoothness of the Curve Criteria: The force curve should be smooth, free from sharp fluctuations or noise. Significance: A smooth curve indicates the absence of external interference during the measurement process, thereby ensuring high-quality data.

### Statistical analysis

Data were collected from five different locations on organoids. A total of 100 force curves were analyzed via the Hertzian (Spherical) model.^5^ The procedure is outlined as follows:

3. Import force curve data: Open the Nanoscope Analysis software and load the collected force curve data, ensuring that each force curve encompasses a complete “Extend” and “Retract” cycle.
4. Select the “Baseline Correction” tool within the software: Adjust the baseline of the non-contact region either automatically or manually to zero, thereby eliminating any offset. Significance: This step removes instrumental drift or noise, ensuring the precision of the force curve analysis.
5. Click “Execute” to extract the indentation segment data.
6. Activate the “Indentation” tool: Fit the compression segment data to calculate the effective elastic modulus. Significance: This process enables the determination of the sample’s elastic modulus through fitting.

**Figure 4:**
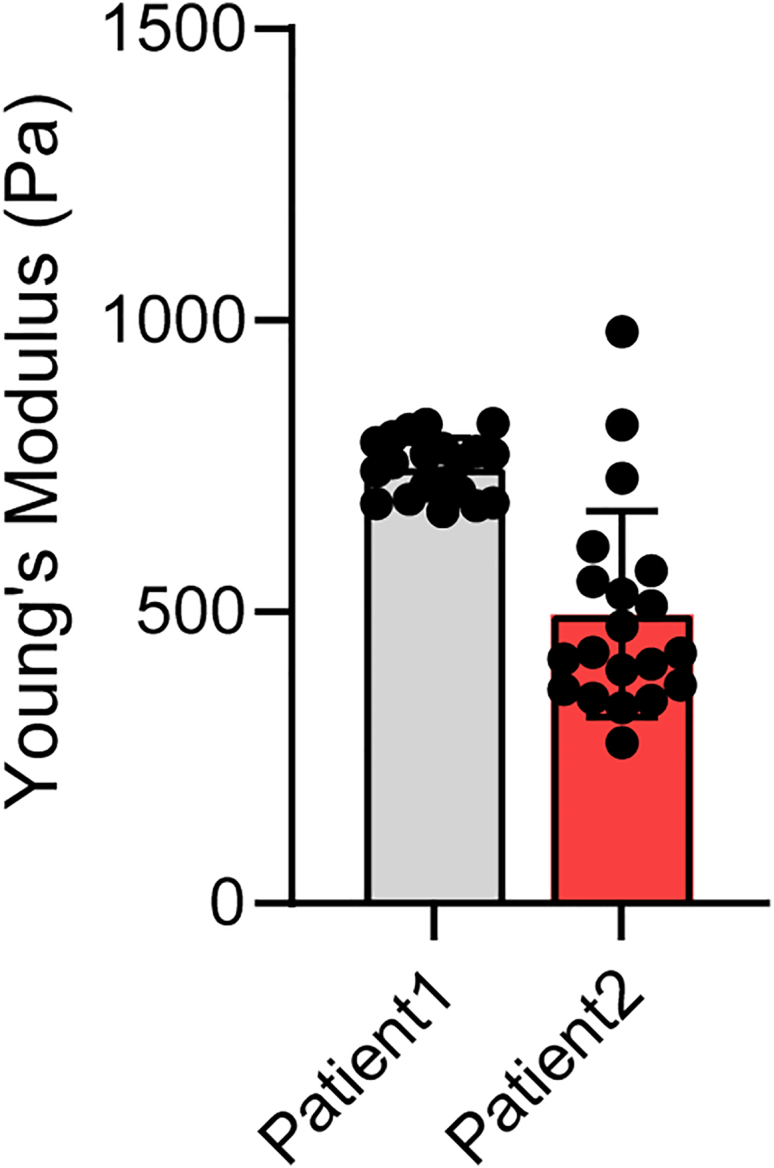
Measurement of Young’s Modulus of colorectal cancer organoids

Column chart is plotted using GraphPad Prism showing average of Young’s Modulus (Figure 4).

## Limitations

• Limited Scope: The methods described in this protocol provide information about the global Young’s Modulus of the organoids but cannot resolve the mechanical properties of specific cell compartments, organelles, or membranes.
• Challenges in Selecting High-Quality Force Curves. Obtaining high-quality force curves is challenging. Only curves with small indentation depths (500–1000 nm), no adhesion effects, and minimal drift are suitable for this method. The Nanoscope data processing software can be used to select such high-quality curves.
• Adhesion Effects were Not Considered. Adhesion effects between the organoids and the probe are not accounted for in this approach. Ensure that adhesion effects in the experimental data are either minimal or entirely absent.

## Troubleshooting

### Problem 1

Strict requirements for cell viability and density in organoid cultures.

### Potential solutions

Reduce the time between specimen collection and transportation to the laboratory. Strengthen cold storage measures.

• Train personnel to reduce the collection and processing time to within 30 min.
• Transport specimens using insulated containers maintained at 4°C.

### Problem 2

Organoids tend to fragment.

### Potential solutions

• Prolong the organoids sedimentation time, for example, up to 3 h.
• Avoid centrifugation, as it can disaggregate organoids and disrupt organoids structures.

### Problem 3

Poor force curves obtained in AFM measurement.

### Potential solutions

Measurements are performed at multiple points and repeated tests are performed to minimize the effect of variability.

### Problem 4

Difficulty in Locating Organoids During Sectioning.

### Potential solutions

• Increase the amount of Trypan blue staining agent appropriately to deepen the color of stained OCT compound. We had confirmed trypan blue-stained OCT compound did not affect the measurement results.
• Pay attention to embed the organoids in the middle when re-embedding, so as to facilitate locating them during sectioning.

### Problem 5

Difficulty in surrounding the organoids with the prepared stained OCT gel to ensure uniform coverage.

### Potential solutions

• To the extent possible, a pipetting gun was utilized to access beneath and alongside the organoids, followed by the slow injection of trypan blue- stained OCT compound.
• After storage at −80°C for 5 min, carefully extract the embedded organoids using a small spoon and transfer it into a pre-prepared tin foil box lined with trypan blue-stained OCT gel. Perform further embedding of the organoids using trypan blue-stained OCT gel for preliminary fixation. Maintain the sample at −80°C for an additional 5 min. Subsequently, re-embed the initially fixed organoids in unstained OCT gel within another pre-prepared tin foil box filled with unstained OCT gel. Adjust the position of the initial embedding block to ensure that the organoids remains centered within the geometric center of the final embedding block during re-embedding. A video of these experimental procedures will be provided as supplementary material.

### Problem 6

Delayed mechanical response or rough surface viscoelastic materials lead to ambiguous force curves at the contact points.

### Potential solutions

• Selecting a probe suitable for the hardness of the sample, such as a soft sample, requires the use of a low elastic constant probe.
• Increasing the acquisition density of the force curve, for example by increasing the number of pixels.

### Problem 7

The scan rate is too high or the number of force curve points is insufficient, resulting in sparse data points. Noise interference or baseline drift causes fluctuations in the force curve. Probe wear or contamination, causing anomalous fluctuations in the force curve. For all these reasons, the curve may not be smooth enough.

### Potential solutions

• Reduce the scan rate (e.g., from 1 Hz to 0.5 Hz) and increase the single point dwell time, increasing the number of sampling points of the force curve.
• The data was pre-processed using a baseline correction function.

## Resource availability

### Lead contact

Further information and requests for resources and reagents should be directed to and will be fulfilled by the lead contact, Yingchi Yang (yangyingchi@ccmu.edu.cn).

### Technical contact

Questions about the technical specifics of performing the protocol should be directed to the technical contact, Tianzhen Zhang (tianzhen_zhang@126.com).

### Materials availability

This protocol does not generate any new material.

### Data and code availability

This study did not generate any unique datasets or code.

## Acknowledgments

This work was supported by 10.13039/100014717National Natural Science Foundation of China grants (no. 8237140092 and no. 82101923), the Capital’s Funds for Health Improvement and Research, CFH (no. 2022-2-1112), the Beijing Municipal Administration of Hospitals’ Youth Program (no. QML20230106), the 10.13039/501100005090Beijing Nova Program (no. 20240484501), the 10.13039/501100004826Beijing Natural Science Foundation (no. 7232035), the Distinguished Young Scholars from Beijing (Friendship Hospital no. yyqcjh2022-4), and the National Key Technologies R&D Program (no. 2015BAI13B09). We appreciate Junpei Yue from Bruker (Beijing) Scientific Technology Co., Ltd. and Zexin Chen from Guangdong Research Center of Organoid Engineering and Technology for their support and efforts with the organoid experiments.

## Author contributions

Conceptualization, T.Z.; methodology, T.Z. and L.M.; investigation, S.L. and Y.C.; writing – original draft, L.M. and Y.C.; writing – review and editing, T.Z., D.T., and Y.Y.; funding acquisition, T.Z., D.T., and Y.Y.; supervision, Z.Z., D.T., and Y.Y.

## Declaration of interests

The authors declare no competing interests.
